# A Real-Time Mechanical Information Acquisition System and Finite Element Prediction Method for Limb Lengthening: A Pilot In Vivo Study

**DOI:** 10.3390/s26061950

**Published:** 2026-03-20

**Authors:** Hao Yang, Tairan Peng, Yuyang Han, Ming Lu, Yunzhi Chen, Zheng Yang

**Affiliations:** 1 Pediatric Orthopaedics Department, National Center for Orthopaedics, Beijing Jishuitan Hospital, Capital Medical University, Beijing 100096, China; 2 Beijing Research Institute of Traumatology and Orthopaedics, Beijing 100035, China; 3 Department of Mechanical Engineering, Tsinghua University, Beijing 100084, China

**Keywords:** limb lengthening, mechanical acquisition system, finite element prediction, in vivo validation, distraction force, soft tissue mechanics, digital twin

## Abstract

In the field of orthopedic surgery, particularly distraction osteogenesis (DO), the mechanical environment plays a decisive role in the quality of bone regeneration and the safety of the soft tissue envelope. The continuous monitoring and accurate prediction of distraction resisting forces (DRF) are critical for preventing soft tissue complications such as nerve ischemia, joint contractures, and mechanical failure of the lengthening device. However, current clinical practice relies heavily on subjective assessment or passive monitoring tools that lack predictive capabilities. To address this gap, this study proposes a comprehensive solution combining a custom mechanical acquisition system with a high-fidelity finite element (FE) prediction method. The system design features a novel “double-ring” sensor interface specifically engineered to decouple axial distraction forces from parasitic bending moments generated by asymmetric muscle tension. Furthermore, a patient-specific FE model utilizing the Ogden hyperelastic constitutive law was derived, explicitly based on the patient’s muscle volume from preoperative CT imaging, to predict the non-linear force evolution. The feasibility and accuracy of the system were validated in a pilot in vivo study using a single ovine model (N=1). To isolate the soft tissue resistance from callus formation, distraction was performed immediately postoperatively up to a total length of 4 cm. Experimental results demonstrated the system’s high linearity ($R^{2}>0.999$) and its ability to capture the characteristic viscoelastic relaxation of living tissues. The FE model successfully predicted the peak distraction forces, showing improved agreement with experimental data at larger distraction magnitudes. By integrating mechanical sensing with predictive modeling, this framework lays the foundation for future closed-loop, patient-specific control in distraction osteogenesis.

## 1. Introduction

### 1.1. Clinical Background: The Biomechanics of Distraction

Distraction Osteogenesis (DO) has revolutionized the treatment of Leg Length Discrepancy (LLD), congenital deformities, and segmental bone defects since its popularization by Ilizarov [1,2]. The fundamental principle, known as the “Law of Tension-Stress,” relies on the gradual traction of living tissue to stimulate neovascularization and osteogenesis. This process is not merely biological but is fundamentally governed by the mechanical environment [3]. While the regenerative capacity of bone under tension is well-documented [2], the process imposes significant mechanical stress on the surrounding soft tissue envelope, including muscles, fascia, nerves, blood vessels, and skin [4,5]. These soft tissues, unlike the mineralized bone, exhibit complex viscoelastic properties. As distraction proceeds, they generate a progressive resistance known as the Distraction Resisting Force (DRF). The magnitude and rate of this force accumulation are critical parameters. Excessive DRF is a primary antagonist in limb lengthening [6]. Clinical studies have shown that high tensile forces can lead to severe complications [7]. Biologically, excessive tension can cause joint subluxation, flexion contractures due to muscle tightness, and permanent nerve damage due to ischemia [8,9]. Mechanically, unpredicted high loads can exceed the yield strength of the lengthening hardware, leading to the jamming of motorized intramedullary nails or the fatigue fracture of external fixator pins [8,10]. Therefore, maintaining the DRF within a safe therapeutic window is crucial for both clinical safety and device integrity [11].

### 1.2. Technological Gaps in Current Monitoring

Despite the clinical importance of DRF, accurate quantification remains a challenge. Traditional assessment methods, such as manual palpation of the limb (“clinical feel”) or patient pain feedback, are subjective, discontinuous, and often reactive rather than proactive [8,12]. Radiographic evaluation, while useful for bone formation [13], provides no direct information on soft tissue tension. In the research domain, instrumented external fixators have been developed to provide objective data. For instance, Mora-Macías et al. developed systems to monitor callus stiffness in various contexts [14,15]. Similarly, Claes et al. and others have instrumented fixators to study interfragmentary movements and fixation stiffness [16,17]. Other notable contributions include the work of Grasa et al. [18] on load transmission and Reifenrath et al. [19] on axial forces in sheep. However, these existing systems often suffer from several limitations. First, regarding the lack of prediction, most systems function solely as data loggers. They record historical force data [20,21] but cannot predict the potential risk of future distraction steps based on the patient’s specific anatomy. This limitation restricts their utility in preoperative planning [22]. Second, coupling errors are a significant issue, as standard external fixators are often subjected to complex loading conditions. Asymmetric muscle tension (e.g., from the triceps surae in tibial lengthening) generates significant bending moments. Many single-sensor designs fail to decouple these moments from the axial distraction force, leading to measurement errors [23,24]. Finally, many studies rely on inaccurate validation models. The majority of validation studies utilize cadaveric (ex vivo) models or simplified bench tests. These approaches fail to replicate the physiological muscle tone, interstitial fluid pressure, and vascular turgor present in living subjects, leading to a significant underestimation of the actual clinical loads [25,26]. Common animal models such as sheep are often used to validate these systems [27,28].

### 1.3. Study Objectives

To address these technological gaps, this paper presents a robust hardware-software framework. This study builds upon the fundamental understanding of fracture healing biology and leverages modern sensing technologies. The specific contributions are: (1) To design a sensor-integrated external fixator with a specific mechanical topology capable of accurately measuring axial loads in the presence of eccentric bending moments; (2) To establish a patient-specific Finite Element (FE) prediction method based on hyperelastic constitutive laws, enabling the forecasting of DRF from preoperative images; and (3) To validate the system’s feasibility and the model’s predictive accuracy through a pilot in vivo animal experiment (N=1), utilizing established protocols, serving as a proof-of-concept for future clinical translation.

## 2. System Design and Mechanical Architecture

### 2.1. Biomechanical Design Requirements

The design of the mechanical acquisition system was driven by specific clinical and engineering requirements appropriate for large animal models (ovine) and potential translation to human use. Unlike general-purpose sensors, a device for limb lengthening must satisfy conflicting constraints. Regarding load capacity versus sensitivity, the system must withstand the peak DRF expected in femoral lengthening (up to 500 N in sheep) while maintaining high sensitivity to detect small force variations (<1 N) during the relaxation phase [29]. In terms of structural rigidity, the integration of sensors must not compromise the overall stiffness of the fixator. The frame must remain rigid enough to prevent shear motion at the osteotomy site, which could inhibit bone formation [30]. Additionally, the system requires decoupling capability to isolate the axial distraction force from parasitic bending moments and shear forces generated by the limb’s weight and muscle contractions [31]. Finally, biocompatibility is essential; all materials in contact with the subject or the sterile field must be biocompatible and capable of withstanding sterilization procedures [1].

### 2.2. Double-Ring Sensor Interface Topology

**Figure 1:**
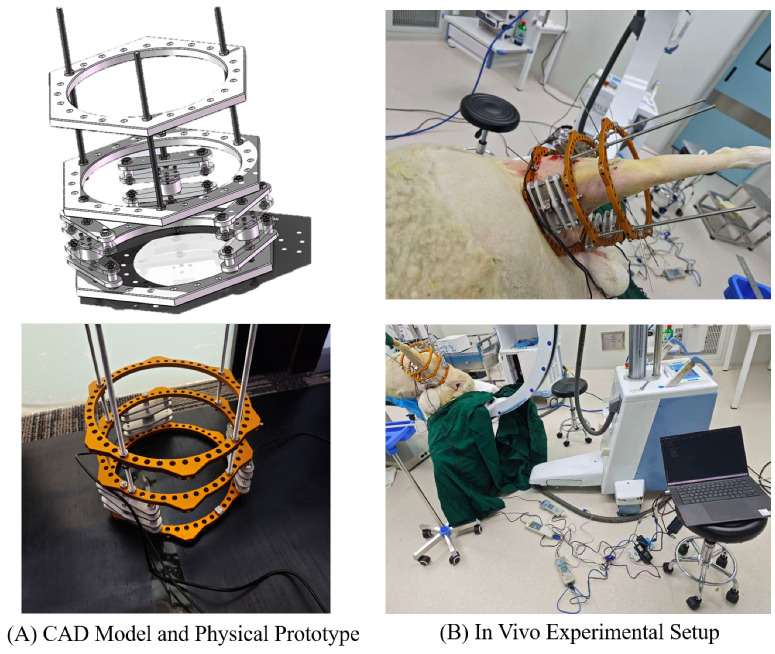
System Design and Experimental Integration. (**A**) CAD design and the manufactured sensor-integrated external fixator prototype. (**B**) The system applied on the ovine model showing the overall experimental setup.

To meet these requirements, a custom external fixator was developed based on the standard Ilizarov configuration [32]. The core innovation is the “Double-Ring Sensor Interface” located at the distal segment of the frame (Figure 1).

#### 2.2.1. Structural Components

The interface consists of two parallel rings machined from aviation-grade 7075-T6 aluminum alloy. This material was selected for its high tensile strength (≈572 MPa) and low density, minimizing the burden on the animal subject. The total weight of the sensing module is approximately 450 g. The rings are separated by three rigid stainless steel standoffs, creating a defined space for sensor integration. The choice of aluminum also ensures compatibility with medical imaging modalities (CT/X-ray) compared to ferromagnetic steel [33].

#### 2.2.2. Sensor Configuration and Moment Decoupling

Embedded between these rings is a triangular array of three compression load sensors (Model DS4-500N). The sensors are distributed equidistantly at 120° intervals. This triangular configuration is not arbitrary; it provides a mechanically determinate structure. In a clinical scenario, the center of resistance of the soft tissues rarely coincides with the geometric center of the fixator. This eccentricity (*e*) generates a bending moment (*M*). If a single sensor were used, this moment would distort the axial reading [18]. However, in our three-sensor configuration, the system acts as a statically determinate plane. By summing the output of the three sensors ($F_{1},F_{2},F_{3}$), the total axial force ($F_{total}$) can be calculated regardless of the load distribution:

(1) $$F_{total}=\sum_{i=1}^{3}F_{i}=F_{axial}$$

Any bending moment manifests as a differential reading between the sensors (e.g., $F_{1}>F_{2}$), but the sum remains invariant to the moment. This design ensures that the measured DRF reflects the true global tissue tension, offering a significant improvement over single-sensor designs [9].

## 3. Theoretical Framework and Prediction Method

While the mechanical system measures the current state, the Finite Element (FE) model provides the capability to predict future loads based on patient anatomy. This predictive capability distinguishes our approach from purely monitoring-based studies.

### 3.1. Continuum Mechanics Formulation

To accurately predict the large deformation behavior of soft tissues, we employed the theory of hyperelasticity within a finite element framework. Let X be the reference position of a material particle and x be its current position. The deformation gradient tensor *F* is defined as:

(2) $$F=\frac{\partial x}{\partial X}$$

The volume change ratio is given by the Jacobian J=det(F). For soft tissues, which are largely composed of water, we assume incompressibility, thus J≈1. The Left Cauchy-Green deformation tensor B, which is objective and strain-measure independent, is given by:

(3) $$B=FF^{T}$$

The principal invariants of B are needed for isotropic constitutive modeling. The first invariant $I_{1}$ is:

(4) $$I_{1}=\mathrm{tr}\left(B\right)=\lambda_{1}^{2}+\lambda_{2}^{2}+\lambda_{3}^{2}$$

where $\lambda_{i}$ are the principal stretches.

### 3.2. Ogden Constitutive Model

Biological soft tissues exhibit highly non-linear, strain-stiffening behavior (J-shaped stress-strain curve). Linear elastic models (Hooke’s Law) are insufficient for capturing this response at large strains. We selected the Ogden phenomenological model due to its superior ability to fit experimental data for biological tissues compared to Neo-Hookean or Mooney-Rivlin models. While higher-order Ogden models (N≥2) can provide a closer fit to complex multi-axial loading data, we employed a first-order (N=1) model for several methodological reasons. First, the primary loading mode in limb lengthening is uniaxial distraction, where a single-term formulation has been shown to sufficiently capture the characteristic stiffening of skeletal muscle [17]. Second, increasing the number of terms introduces additional independent material parameters, which significantly increases the risk of non-unique parameter identification and over-fitting given our single-subject experimental input. A first-order model maintains high computational efficiency and provides a more robust, stable solution for clinical predictive tools where fewer patient-specific parameters are available for calibration. The Strain Energy Density Function (SEDF), Ψ, for a first-order Ogden model is expressed in terms of the principal stretches:

(5) $$\Psi =\frac{\mu }{\alpha }\left(\lambda_{1}^{\alpha }+\lambda_{2}^{\alpha }+\lambda_{3}^{\alpha }-3\right)+\frac{1}{D_{1}}{\left(J-1\right)}^{2}$$

where μ is the shear modulus, α is the strain hardening exponent, and $D_{1}$ is the incompressibility parameter. The principal Cauchy stresses $\sigma_{i}$ are derived from the SEDF:

(6) $$\sigma_{i}=\lambda_{i}\frac{\partial \Psi }{\partial \lambda_{i}}-p=\mu \lambda_{i}^{\alpha }-p$$

where *p* is the hydrostatic pressure Lagrange multiplier used to enforce the incompressibility constraint.

### 3.3. Computational Implementation

**Figure 2:**
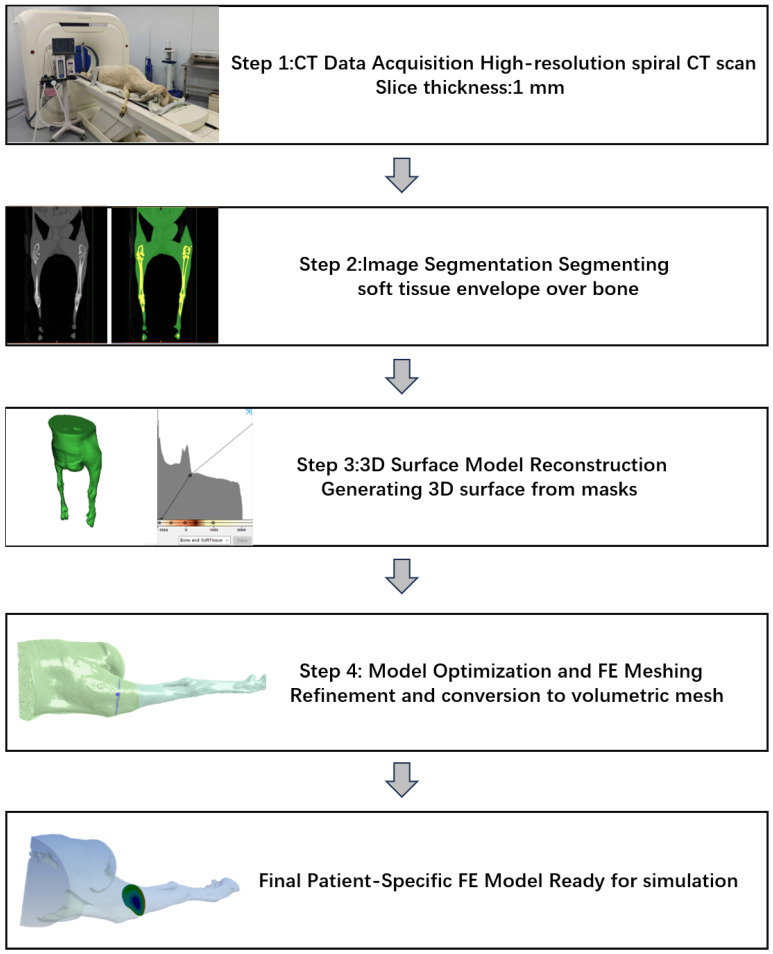
Computational workflow: (1) CT Imaging → (2) Soft Tissue Segmentation → (3) 3D Reconstruction → (4) FE Mesh Generation.

**Figure 3:**
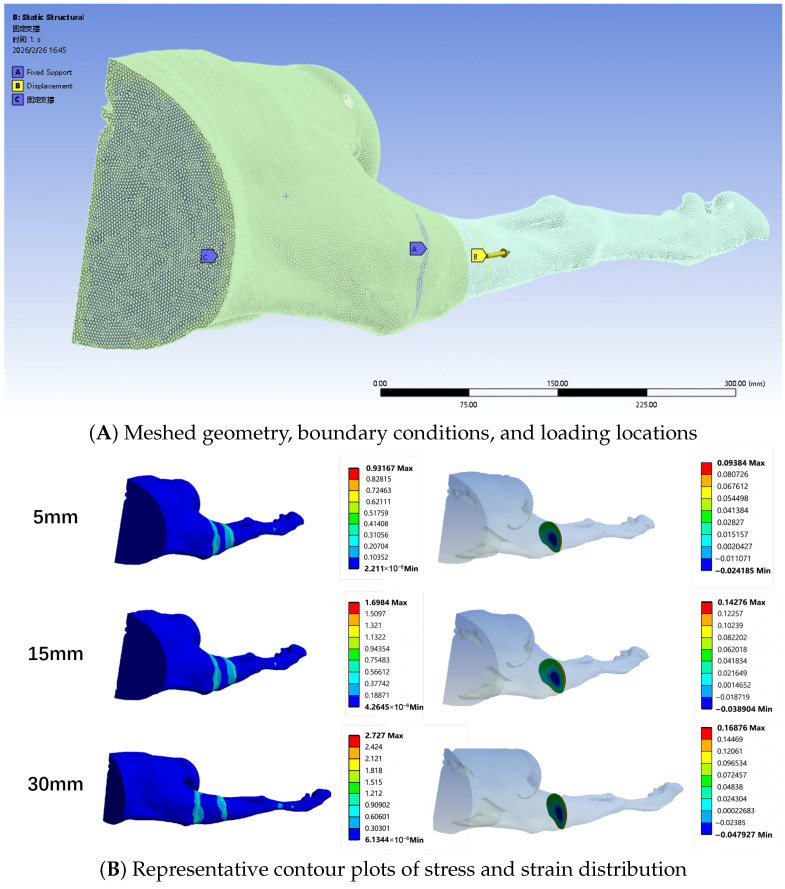
Finite Element Model Setup and Representative Results. (**A**) The meshed geometry of the ovine hind limb soft tissue envelope. The applied boundary conditions include proximal fixed supports (labeled A and C) and a distal displacement load (labeled B) directed along the anatomical axis to simulate the distraction process. (**B**) Representative contour plots illustrating the internal stress (left column) and strain (right column) distributions within the soft tissue at progressive distraction steps of 5 mm, 15 mm, and 30 mm.

The prediction pipeline begins with high-resolution spiral CT scans of the ovine hind limbs using a Sinovision CT scanner (Sinovision, Beijing, China). The scanning parameters were set to 120 kV and 253 mA, with a slice thickness of 1.0 mm. The scan covered the region of the pelvis and both hind limbs, yielding a total of 815 axial slices with a 512 × 512 matrix. The process involves several key steps. First, regarding reconstruction, unlike traditional bone-centric models, our reconstruction focused on the Soft Tissue Envelope (STE). Recognizing that the skeletal muscle is the primary source of passive tension contributing to DRF, the segmentation process was performed using Mimics medical imaging software (Materialise, Leuven, Belgium) to prioritize the accurate reconstruction of muscle volume. The major muscle groups along with fascia and skin bridging the osteotomy gap were segmented and converted into a volumetric mesh (Figure 2). Recognizing that the skeletal muscle is the primary source of passive tension contributing to DRF, the segmentation process prioritized the accurate reconstruction of muscle volume. The major muscle groups along with fascia and skin bridging the osteotomy gap were segmented in medical imaging software and converted into a volumetric mesh (Figure 2). Second, for meshing, the geometry was discretized using 10-node tetrahedral elements (C3D10). Convergence was achieved at an element size of 2 mm, resulting in a mesh of approximately 250,000 elements. Third, regarding boundary conditions, the proximal end of the limb model was fully constrained (Fixed Support). A remote displacement boundary condition was applied to the distal cross-section, simulating the distraction steps of up to 40 mm along the anatomical axis (Figure 3A). Finally, material parameters for the first-order Ogden model were defined as follows: shear modulus μ=700 kPa, strain hardening exponent α=20, and incompressibility parameter $D_{1}=0.049$ Pa^−1^. While soft tissues are often idealized as perfectly incompressible, a small non-zero value for $D_{1}$ was utilized in the FE solver (Ansys Workbench) to ensure numerical convergence while maintaining a high bulk-to-shear modulus ratio, effectively simulating the nearly-incompressible behavior of skeletal muscle.

## 4. Validation Experiments and Results

### 4.1. In Vitro Metrological Calibration

**Figure 4:**
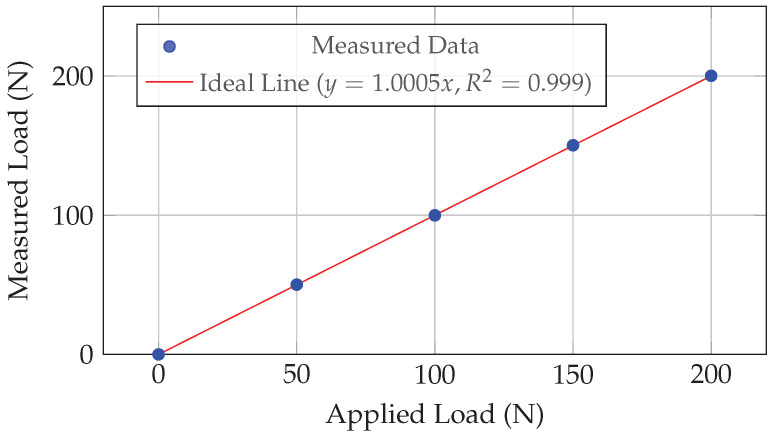
In vitro calibration curve showing the high linearity of the acquisition system. The linear fit indicates $R^{2}>0.999$.

Before the animal study, the acquisition system underwent rigorous benchtop calibration to quantify its metrological performance using standard weights (Class F1) to apply known axial loads. During the experimental measurements, the force readings exhibited high stability with minimal signal fluctuations; the electronic noise floor was maintained well within ±0.15 N through the use of a 24-bit AD conversion module and a low-pass Butterworth filter. The system demonstrated excellent linearity across the 0–200 N range, with a coefficient of determination $R^{2}>0.999$ (Figure 4). Hysteresis was assessed by performing a loading and unloading cycle; the maximum hysteresis error was found to be less than 0.1%, indicating minimal energy dissipation in the sensor structure. Stability was verified by applying a constant load of 100 N for 1 h to measure zero-point drift, which was below 0.2 N, confirming the structural stability. Measurement variability was strictly controlled within ±0.5 N across repeated trials. Finally, following the standard Guide to the Expression of Uncertainty in Measurement (GUM), the expanded uncertainty (k=2) was calculated to be ±0.35 N.

### 4.2. Pilot In Vivo Animal Experiment

To validate the feasibility of the system in a physiological environment, a pilot study was conducted on one skeletal mature sheep. The study protocol was approved by the Institutional Animal Care and Use Committee.

#### 4.2.1. Preoperative Preparation and Surgery

**Figure 5:**
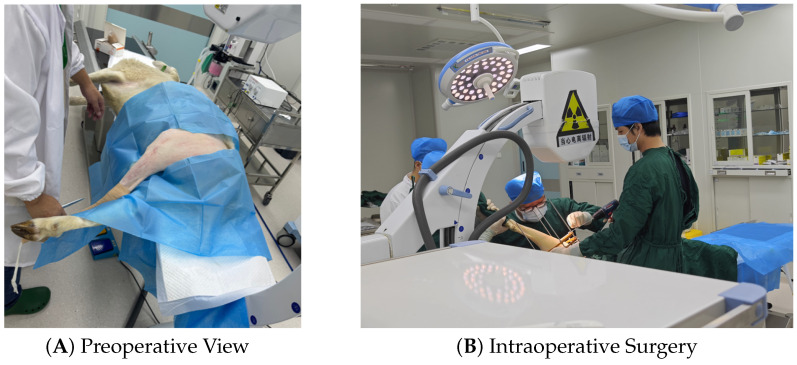
Surgical procedure validation. (**A**) Preoperative photograph of the ovine subject positioned for surgery. (**B**) Intraoperative photograph showing the surgeon performing the procedure.

To validate the feasibility of the system in a physiological environment, a pilot study was conducted on a skeletal mature male Hu sheep (*Ovis aries*). The subject was 14 months old with a body weight of 70 kg. The animal was fasted for 24 h prior to surgery. Preemptive analgesia (Meloxicam, 0.5 mg/kg) and prophylactic antibiotics (Cefazolin, 20 mg/kg) were administered intravenously. Under general anesthesia (Propofol induction, Isoflurane maintenance), a 3 cm lateral incision was made to expose the mid-shaft femur. The custom sensor-integrated fixator was mounted using 2.0 mm external fixator pins proximally and distally. A transverse mid-shaft osteotomy was performed using an oscillating saw under continuous saline irrigation (Figure 5). Under general anesthesia (Propofol induction, Isoflurane maintenance), a 3 cm lateral incision was made to expose the mid-shaft femur. The custom sensor-integrated fixator was mounted using 2.0 mm external fixator pins proximally and distally. A transverse mid-shaft osteotomy was performed using an oscillating saw under continuous saline irrigation (Figure 5).

#### 4.2.2. Distraction Protocol

In bone elongation, the Total Distraction Force (TDF) is composed of the force necessary to distract callus tissue (Callus Distraction Force, CDF) and the force to overcome soft tissue resistance (Distraction Resisting Force, DRF). To specifically characterize the soft tissue envelope’s contribution and avoid the interference of callus formation (CDF), the distraction protocol was initiated immediately postoperatively, bypassing the traditional latency period. Acute distraction steps were applied to the limb, reaching a total elongation of 4 cm (40 mm). Real-time force data was recorded continuously during distraction and for a 2-min relaxation period after each step to capture the static equilibrium force.

### 4.3. Results: Single-Subject Data vs. Prediction

**Figure 6:**
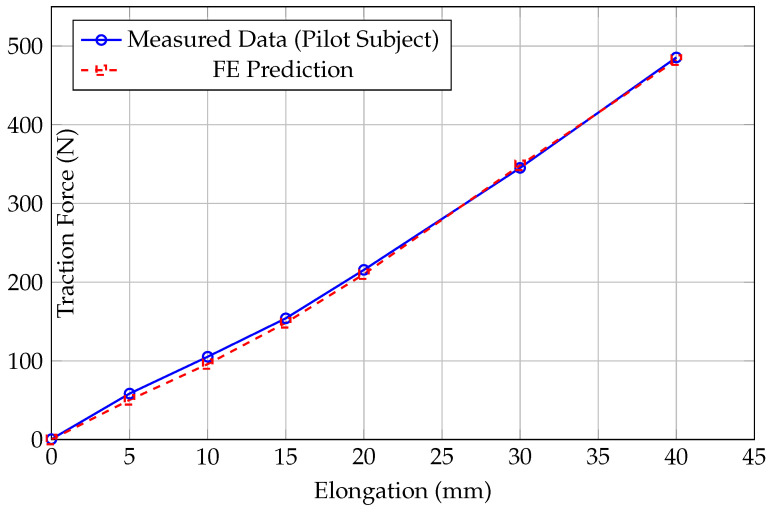
Validation results for the pilot study (N=1) up to 40 mm. The measured force (Blue) exhibits the characteristic non-linear stiffening of soft tissue. The FE prediction (Red) converges closely with the experimental data as the lengthening distance increases, particularly at 40 mm.

**Figure 7:**
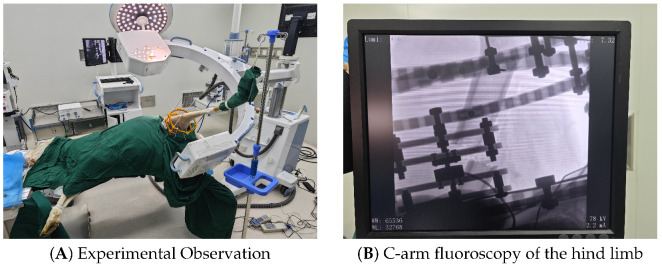
Radiographic validation. (**A**) Observation of the limb during the validation phase. (**B**) Following distraction protocol, confirming the generated distraction gap and the response of the soft tissue envelope.

The in vivo measurements from the pilot subject revealed a distinct non-linear increase in DRF (Figure 6). The force curves exhibited significant stress relaxation immediately after distraction, confirming the viscoelastic nature of the living tissue. Notably, the agreement between the FE prediction and the measured data improved with increased distraction length. To verify the prediction method, the FE simulation results were compared with the experimental data up to 40 mm. As illustrated in Figure 6, the non-linear J-shaped curve is well captured. Representative contour plots illustrating the stress and strain distribution within the soft tissue envelope during progressive distraction steps (5 mm, 15 mm, 30 mm) are shown in Figure 3B. At 40 mm, the measured force reached 485.5 N, while the FE model predicted 482.1 N, demonstrating a high degree of convergence at large deformation states. Table 1 summarizes the accuracy at each distraction step. Finally, radiographic verification was performed to ensure the validity of the distraction gap (Figure 7).

## 5. Discussion and Conclusions

This study developed and validated a comprehensive system for the mechanical assessment of limb lengthening. The results demonstrate the system’s ability to operate in a real-world clinical environment, capturing critical biomechanical phenomena. The force evolution observed in our study, characterized by acute peaks followed by significant exponential decay, aligns with the temporal force patterns described by Leong et al. [11], who emphasized the rapid viscoelastic relaxation of soft tissues immediately post-distraction. Furthermore, the correlation we established between the distraction gap and the resisting force mirrors the spatial characterization of the regenerate environment reported by Richards et al. [7], confirming that mechanical tension accumulates non-linearly as the distraction distance increases. Quantitatively, the peak distraction resisting forces (reaching approximately 485 N at 4 cm) and the stiffness of the soft tissue envelope fall specifically within the physiological ranges reported in similar ovine models. Our data is consistent with the findings of Vetter et al. [17], who documented the substantial contribution of the lengthened muscle-tendon unit to passive tension, and Steiner et al. [34], who established baseline mechanical properties for the callus and soft tissue complex in sheep. By reproducing these specific mechanical behaviors, our system validates its accuracy and utility for in vivo applications. Unlike previous wireless monitoring systems which focused primarily on hardware implementation, the present work integrates FE-based predictive modeling for proactive mechanical assessment.

1. **Superior Measurement Stability**: The “double-ring” sensor interface offers a distinct advantage over traditional monitoring methods regarding data stability. Previous systems relying on single-point sensors or strain gauges on fixator pins are highly susceptible to “cross-talk” errors caused by bending moments, often resulting in noisy data when the subject moves [18]. In contrast, our triangular three-sensor configuration creates a mechanically determinate plane that inherently decouples axial forces from bending moments. This design ensures that the acquired DRF data remains stable and accurate, even in the presence of eccentric loading caused by the animal’s posture or activity.
2. **Clinical Relevance for Large Lengthening**: Clinically, surgical indication for limb lengthening is typically reserved for patients with a Leg Length Discrepancy (LLD) greater than 2 cm. As demonstrated in our results, the DRF increases non-linearly; beyond 2 cm or 3 cm of lengthening, the tension generated by the soft tissue envelope escalates rapidly. This high tension poses a significant risk for the mechanical failure of the lengthening device, particularly for Intramedullary Nails (IMN), which may jam or fracture under excessive load [8]. Our FE model demonstrated superior predictive accuracy at these larger distraction distances (30–40 mm), precisely where the clinical risk is highest. This capability suggests that the proposed method is well-suited for preoperative planning in cases requiring substantial lengthening, potentially preventing hardware failure by predicting unsafe load thresholds.
3. **Evaluation of the Acute Distraction Protocol**: It is important to acknowledge the limitations of the acute distraction protocol employed in this pilot study. By applying immediate, large-step distraction, the experiment does not account for the biological phenomenon of stress relaxation via tissue regeneration and growth (neohistogenesis) that occurs during clinical gradual lengthening. However, this experimental design was intentional and advantageous for the specific validation goals of this study. It effectively eliminated the confounding variable of callus formation (Callus Distraction Force), ensuring that the measured forces represented the pure mechanical response of the soft tissue envelope. This isolation was crucial for validating the accuracy of the hyperelastic FE model based on muscle volume.
4. **Predictive Accuracy and Muscle Volume**: The FE model, utilizing a patient-specific Ogden constitutive law, accurately forecast distraction forces with an RMSE of 6.45 N. The high fidelity of the prediction validates the modeling strategy of prioritizing muscle volume reconstruction, confirming that skeletal muscle volume is the dominant factor determining the magnitude of Distraction Resisting Force. This finding suggests that future patient-specific models can focus primarily on muscle segmentation to achieve clinically relevant predictions.
5. **Potential Clinical Significance**: This framework represents an exploratory step towards more precise distraction osteogenesis. Current clinical decision-making often relies on subjective markers, such as manual palpation or patient pain, which can be reactive. By integrating mechanical sensing with predictive modeling, this pilot study indicates the potential to transform the treatment approach from a generalized empirical protocol to a more individualized regime. Such a system could eventually help in defining a therapeutic safety window, potentially assisting surgeons in anticipating complications while allowing for future optimization of the distraction rate based on individual physiological tolerance.

Future work will prioritize increasing the sample size of the in vivo study to better account for biological variation and ensure the robustness of the findings across different subjects. Additionally, future efforts will focus on integrating feedback control to realize a closed-loop intelligent external fixator [35]. Other researchers have also highlighted the potential of such systems for various applications [21,36].

## Figures and Tables

**Table 1:** Comparison of measured vs. predicted forces for the single-subject pilot study.

Distraction Step	Measured Force (N)	Predicted Force (N)	Absolute Error (N)
5 mm	58.4	50.32	8.08
10 mm	105.2	95.89	9.31
15 mm	154.0	148.35	5.65
20 mm	215.6	210.10	5.50
30 mm	345.2	348.50	3.30
40 mm	485.5	482.10	3.40
Overall RMSE			6.45 N

## Data Availability

The datasets generated during and/or analyzed during the current study are available from the corresponding author on reasonable request.
